# COMPARING HEALTH SYSTEM–BASED AND COMMUNITY-BASED DEMENTIA CARE: THE DEMENTIA CARE (D-CARE) STUDY

**DOI:** 10.1093/geroni/igae098.1466

**Published:** 2024-12-31

**Authors:** David Reuben, Jenny Summapund, Alan Stevens, Thomas Gill, Peter Peduzzi, Rebecca Galloway, Martha Carnie

**Affiliations:** University of California Los Angeles David Geffen School of Medicine, Los Angeles, California, United States; University of California Los Angeles David Geffen School of Medicine, Los Angeles, California, United States; Baylor Scott & White Health, Belton, Texas, United States; Yale University, New Haven, Connecticut, United States; Yale University, New Haven, Connecticut, United States; The University of Texas Medical Branch, Galveston, Texas, United States; Brigham and Women’s Hospital, Boston, Massachusetts, United States

## Abstract

Despite the advent of new medications for treatment of dementia, the clinical care of those affected remains a challenge. Though several community-based and health system-based approaches have been developed to provide comprehensive care for patients living with dementia and their caregivers, the relative effectiveness of these approaches is unknown. The Dementia Care (D-CARE) Study is a pragmatic randomized trial comparing the effectiveness of three dementia care approaches at four clinical trial sites (CTS) over 18 months: 1) by nurse practitioners or physician assistants within a health care system, 2) by social workers or nurses at community-based organizations, or 3) usual care. D-CARE illustrates advantages (e.g., facilitation of recruitment with over 93% receiving active intervention) and disadvantages (e.g., CTS health systems needed to implement two interventions; because both active interventions have prior demonstrated effectiveness, the differences are likely to be smaller, requiring a larger sample size to have sufficient power) of comparative effectiveness research. The pragmatic design presented obstacles including funding for clinical positions, integrating interventions into workflows, and potential threats to fidelity; but the real-world settings will facilitate dissemination. COVID-19 introduced additional barriers to recruitment and retention. Strong commitment by study leaders, adaptability of research staff, and continuous engagement of patient and stakeholders at the CTS contributed to D-CARE’s success in enrolling 2,176 participants and high retention rates. D-CARE applied principles of community-based participatory research to continually refine materials to the study’s needs. D-CARE’s adaptations and successes demonstrate viable strategies for sustainability and scalability in real-world practices.

